# Continuous-Wave Fiber Cavity Ringdown Pressure Sensing Based on Frequency-Shifted Interferometry

**DOI:** 10.3390/s18041207

**Published:** 2018-04-16

**Authors:** Yiwen Ou, Chunfu Cheng, Zehao Chen, Zhangyong Yang, Hui Lv, Li Qian

**Affiliations:** 1 Hubei Collaborative Innovation Center for High-Efficiency Utilization of Solar Energy, Hubei University of Technology, Wuhan 430068, China; ouyiwen@hbut.edu.cn (Y.O.); chenzehao@hbut.edu.cn (Z.C.); yangzhangyong@hbut.edu.cn (Z.Y.); lvhui@hbut.edu.cn (H.L.); 2 School of Science, Hubei University of Technology, Wuhan 430068, China; 3 Department of Electrical and Computer Engineering, University of Toronto, Toronto, ON M5S 3G4, Canada; l.qian@utoronto.ca

**Keywords:** fiber cavity ringdown, frequency-shifted interferometry, pressure sensing, sensitivity

## Abstract

We present a continuous-wave fiber cavity ringdown (FCRD) pressure-sensing method based on frequency-shifted interferometry (FSI). Compared with traditional CRD or FCRD techniques, this FSI-FCRD scheme deduces pressure by measuring the decay rate of continuous light exiting the fiber ringdown cavity (RDC) in the spatial domain (i.e., the CRD distance), without the requirement for optical pulsation and fast electronics. By using a section of fiber with the buffer layer stripped in the fiber RDC as the sensor head, pressures were measured within the range from 0 to 10.4 MPa. The sensitivity of 0.02356/(km∙MPa) was obtained with a measurement error of 0.1%, and the corresponding pressure resolution was 0.05 MPa. It was found that the measurement sensitivity can be improved by enlarging the interaction length of the sensor head. The results show the proposed sensor has the advantages of simple structure, low cost, high sensitivity, and high stability in pressure detection.

## 1. Introduction

The role of pressure sensors is becoming increasingly prominent in the fields of energy, chemical industry, aerospace, transportation, large civil engineering, etc. Compared with traditional electrical or mechanical pressure sensors, fiber-optic pressure sensors have many outstanding advantages, such as light weight, high sensitivity, high resolution, fast response, and anti-electromagnetic disturbance [1,2,3,4,5].The most popular sensors are based on fiber Bragg gratings (FBGs) and Fabry–Perot interference (FPI). FPI-based sensors measure pressures by monitoring the variation of cavity length, resonant wavelength, or phase shift from the interference spectra caused by external pressures. Many efforts have been devoted to improving the sensitivity of FPI-based pressure sensors by electrical arc discharging, femtosecond laser micromachining, or polymer coating on a bare fiber [6,7,8,9], but the fabrication processes are usually complex, expensive, or poorly reproducible, which hinder their mass production and applications in engineering. The principle of FBG-based pressure sensors is the fact that the Bragg wavelength changes with the variation of pressure. But due to cross sensitivity to other parameters (such as temperature, strain, and vibration) and low sensitivity pressure, FBG-based pressure sensors also require specific mechanical devices and sensitization measures [10,11,12,13]. Therefore, it is crucial to explore new methods for developing fiber pressure sensors that are simple, inexpensive, and highly sensitive.

As a sensitive technique, powerful in measuring weak optical losses, the cavity ringdown (CRD) technique has attracted considerable attention during the past three decades [14]. The CRD technique measures the intensity of the decay rate of an optical pulse exiting the ringdown cavity, rather than the intensity decay of the light transmitted through the test sample like conventional direct or multi-pass optical spectroscopic techniques. The small absorption loss induced by the sample can be monitored by measuring the change of 1/e intensity decay time (i.e., the CRD time). The CRD technique has a long light–sample interaction length and immunity to light source fluctuations, which makes it offer superior sensitivity. However, traditional CRD techniques suffer from the disturbances of inconvenient mirror alignments and delicate coating or polishing of high-finesse ringdown cavities [15,16,17]. In order to overcome these obstacles, a fiber cavity ringdown (FCRD) technique, which employs a fiber loop formed by two fiber couplers as the ringdown cavity(RDC), was first proposed in 2001 [18], and it has rapidly aroused the interest of researchers. In 2004, Wang explored FCRD for pressure measurement and obtained a sensitivity of 0.0432/(s∙Mpa) [19]. More recently, Yang et al. developed this technique for weight-in-motion detection and measured the velocity of vehicles by applying sensing forces on a micro-bending sensor head [20]. Also, Yang et al. reported static ice pressure detection of hydraulic structures based on FCRD technique, and a high sensitivity of 0.0098/(s∙KPa) was attained [21]. Despite these successes, pulsed light is still needed in the fiber RDC for an observable CRD event in FCRD, as well as in CRD. Consequently, an expensive pulsed light source (or a pulse modulator), a fast photoelectric detector, and a high-speed data acquisition card (DAQ, or oscilloscope) are still necessary [22].

Here, we propose to utilize frequency-shifted interferometry fiber cavity ringdown (FSI-FCRD) technique for pressure sensing. FSI-FCRD combines the highly-sensitive virtues of both FSI and FCRD, and thus, it has a highly-sensitive nature to pressure without the request for specific sensitization, unlike FBG-based sensors. In contrast with CRD and FCRD schemes, FSI-FCRD uses continuous light without the need for any optical pulses and fast electronics. Furthermore, FSI-FCRD obtains a CRD transient as a function of the distance traveled by the continuous light in the fiber RDC by operating Fourier transform on the interference signal. Therefore, FSI-FCRD is a continuous-wave (CW) spatial-domain FCRD technique. It measures the decay rate of continuous light in the spatial domain instead of measuring the decay rate of an optical pulse in the time domain as in CRD and FCRD. Additionally, due to the nature of same-path interference and the differential detection utilized to offset direct current (DC) noise of the interference signal, the FSI-FCRD scheme has high signal-to-noise ratio and high stability. FSI-FCRD has been successfully applied in monitoring fiber bend loss, solution refractive index, magnetic field, and gas concentration [22,23,24,25]. However, to the best of our knowledge, there are no publications that have reported the application of this technique in pressure detection.

We fabricated a continuous-wave (CW) fiber cavity ringdown pressure sensor by using a FSI-FCRD scheme. In the scheme, a CW light source, a slow detector, and a slow DAQ were used to monitor the CRD transient of continuous light in the spatial domain. The sensor head was formed by a fraction of single-mode fiber (SMF) in the fiber RDC, which is easy to fabricate, low-cost and repeatable, unlike most FPI-based sensors. By applying different weights on a section of fiber in the fiber ringdown cavity, pressures were successfully measured in the range of 0–10.4 Mpa. The obtained sensitivity was 0.02356/(km∙MPa) with a baseline stability of 0.1% and a resolution of 0.05 MPa, which is better than some results reported by traditional FCRD techniques. This is the first time FSI-FCRD has been used to detect pressure, and the results show that the new type of pressure sensor explored in this work would be a superior alternative to pressure measurement in the aspects of low cost, simple structure, high sensitivity, and high stability.

## 2. Operating Principle

**Figure 1:**
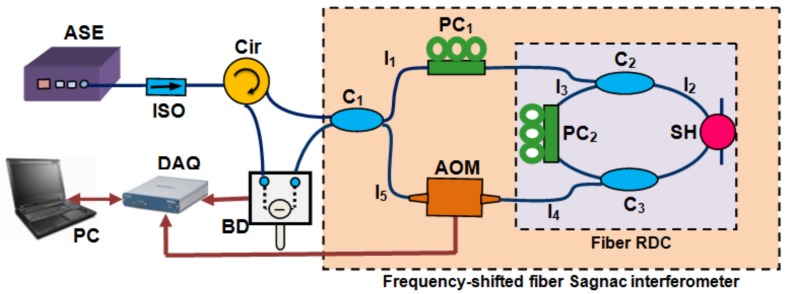
Schematic of the frequency-shifted interferometry fiber cavity ringdown (FSI-FCRD) pressure-sensing system. ASE: amplified spontaneous emission source; ISO: isolator; Cir: circulator; C_1_: 3 dB fiber coupler; C_2_ and C_3_: 99.5/0.5 fiber couplers; PC_1_ and PC_2_: polarization control; SH: sensor head; AOM: acousto-optic modulator; BD: balanced detector; DAQ: data acquisition card; and PC: personal computer.

The schematic of the proposed FSI-FCRD system for pressure measurement is shown in Figure 1. It is essentially an asymmetric frequency-shifted fiber Sagnac interferometer, whose principle has been elaborated in [26]. By connecting the two ports of a fiber coupler (C_1_ in Figure 1), the interferometer is formed with an asymmetrically-inserted frequency shifter (here, an acousto-optic modulator (AOM) is used) and a fiber RDC. The fiber RDC consists of two fiber couplers (C_2_ and C_3_) and two sections of single-mode fiber. After the CW light from a broadband light source is launched into the interferometer loop, two counter-propagating lightwaves will circulate in the fiber RDC. After each roundtrip in the cavity, small fractions of the two lightwaves leak out from the cavity and return to the coupler C_1_. If the coherence length of the light source is shorter than the cavity length, the two lightwaves will interfere at the coupler C_1_ after completing the same number of roundtrips. The differential interference signal between the two output ports of the interferometer can be expressed as [23,24]

(1) $$\Delta I\propto \sum_{m=0}^{\infty }I_{m}\cos\left[2\pi \frac{n\left(mL+L_{0}\right)}{c}f\right]=\sum_{m=0}^{\infty }I_{m}\cos\left(2\pi F_{m}f\right)$$

where *L* = *l*_2_ + *l*_3_ is the ringdown cavity length, *L*_0_= *l*_1_ + *l*_2_ + *l*_4_−*l*_5_ is a constant with *l_i_* (*I* = 1, 2, 4, 5) being the fiber lengths defined in Figure 1, *n* is the effective refractive index of the mode of the single-mode fiber, *f* is the frequency shift caused by the AOM, and *c* is the light speed in vacuum. *F_m_* = *n*(*mL* + *L*_0_)/*c* is the oscillation frequency, which has a linear relationship with the roundtrip number *m*. *I_m_* is the intensity of the interferential light after *m* roundtrips in the RDC [23]:

(2) $$I_{m}=I_{0}\cdot \exp(-m\alpha_{c})=I_{0}\cdot \exp(-\frac{l}{L}\alpha_{c})$$

where *I*_0_ is a constant, *l* = *mL* is the distance circulated by the light in the cavity, and *α_c_* is the background cavity loss per roundtrip, including the fiber couplers’ insertion loss, fiber transmission loss, and fiber splicing loss.

From Equations (1) and (2) Δ*I* is a summation of sinusoids, and *Im* is an exponential decay function of the distance *l*. If we linearly sweep the frequency shift *f* and perform fast Fourier transform (FFT) on Δ*I*, the Fourier spectrum of Δ*I* will include many exponential decay Fourier peaks located at *F_m_*. When frequency *F_m_* is converted to distance *l_d_* using the equation *l_d_* = *l* + *L*_0_* = cF_m_/n*, the Fourier spectrum of Δ*I* is essentially a CRD transient in the spatial domain [23]. That is, the spatial-domain CRD decay transient is obtained by performing FFT on Δ*I*. When the intensity *I_m_* decreases to 1/*e* of the initial intensity (*I*_0_) observed by the detector, the distance traveled by light in the RDC can be termed as the ringdown distance Λ*_0_*, which is similar to the ringdown time in the traditional CRD and FCRD techniques, and it can be described as [23]:

(3) $$\Lambda_{0}=\frac{L}{\alpha_{c}}$$

When an external force *F* is applied vertically on a section of the fiber in the RDC, the pressure *P* equals the force *F* divided by the area of contact *S* (i.e., *P* = *F*/*S*). Furthermore, a pressure-induced loss *α_s_* occurs and leads to a change in the ringdown distance Λ:

(4) $$\Lambda =\frac{L}{\alpha_{c}+\alpha_{s}}$$

where *α_s_* = *ξSdP*, *ξ* is the pressure-induced attenuation coefficient, and *d* is the length of the fiber that has direct interaction with the applied pressure [19], which represents the actual length of the sensor head. Combining Equation (3) with Equation (4), we have

(5) $$(\frac{1}{\Lambda }-\frac{1}{\Lambda_{0}})=\frac{\Delta \Lambda }{\Lambda \Lambda_{0}}=\frac{\alpha_{s}}{L}=\frac{\xi sd}{L}P=kP$$

where *k = ξSd/L* is a constant. Thus, one can deduce the applied pressure by observing the change between the ringdown distance with the pressure applied (Λ) and the ringdown distance without pressure applied (Λ_0_), for a given FSI-FCRD pressure sensor. There is a linear relationship between the variation (1/Λ − 1/Λ_0_) and the applied pressure. From Equation (5), the minimum detectable pressure, that is, the pressure resolution of the system *P*_min,_ can be estimated from the following equations:

(6) $$P=\frac{\Delta \Lambda }{k\Lambda \Lambda_{0}}$$

(7) $$P_{\min}=\frac{\delta \Lambda }{k\Lambda \Lambda_{0}}\approx \frac{\delta \Lambda }{k{\bar{\Lambda }}^{2}}$$

where *δ*Λ and $\bar{\Lambda }$ are one standard deviation of the CRD distances measured repeatedly and the average CRD distance under a given pressure, respectively. Because the constant *k = ξSd/L* can represent the sensitivity of the pressure-sensing system, one can tailor the sensitivity by adjusting the interaction length of *d*. Obviously, the resolution is proportional to the pressure sensitivity. In addition, the two counter-propagating lightwaves in the Sagnac loop travel along the same path, and the two interference signals are detected and subtracted by the balanced detector (BD).Thus, it removes the influences of external disturbances, such as draw, pressure, or twist [22,27] and makes the FSI-based sensing system very steady [28]. Moreover, compared to traditional CRD or FCRD pressure sensors, this system employs continuous light, requiring no pulsation or high-speed detection/data acquisition.

## 3. Experiments and Results

**Figure 2:**
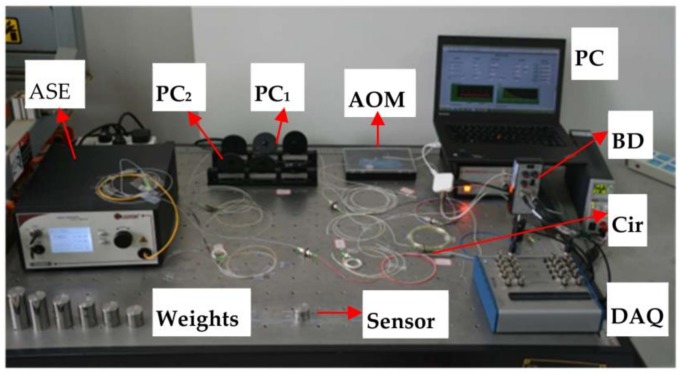
The experimental setup of the fiber pressure detection.

The FSI-FCRD pressure detection system in our laboratory is demonstrated in Figure 2. The fiber RDC constructed by a pair of 99.5:0.5 fiber couplers, had a length of ~ 60 m. An amplified spontaneous emission (ASE) source (Connet Fiber Optics Co., Ltd., Shanghai, China, AS3210-BB2) was used as a broadband light source, with the output power set to 8 mW. The light from the ASE was launched into the Sagnac interferometer formed by a 50/50 fiber coupler C_1_ after passing through an isolator (ISO). The AOM (Brimrose, Maryland, MD, USA, AMM-100-20-25-1550-2FP)was swept from 90 to 110 MHz at a step size of 0.02 MHz, providing a spatial resolution of ~10 m and a sensing range of ~5200 m (>70 roundtrips). The two interference signals of the Sagnac interferometer were converted into electrical signals and subtracted by a BD (New Focus) to eliminate the DC noise, and then, the output of the BD was recorded by a DAQ (NI USB-6361). The maximum sampling rate of the DAQ was set to only 100 kS/s, which was much lower than that used in many traditional CRD or FCRD techniques [23,27,29]. A Labview program was developed for data processing, real-time display, and controlling the synchronization between the AOM and DAQ. Two polarization controllers (PC_1_ and PC_2_) were used to adjust the polarization state and improve the visibility of the interference fringes.

The pressure sensor head was fabricated by two sections of single-mode fiber with the buffer layer stripped, one of which was from the fiber in the RDC, and the other of which was from an individual piece of SMF. The two sections of fiber were fixed on the optical platform and kept parallel to each other. The pressure force *F* was provided by a series of customized cylindrical weights with a diameter of 3 cm and a standard weight. Each cylindrical weight was placed across the top of the sensor head in turn. The interaction length between the sensor head and the weight was set to ~15 mm, and the pressure was varied using the precision weights. Because the contact area could be considered to be a rectangular shape with the width *w* of one-tenth of the fiber cladding diameter (125 m) [19] and the length *d* of 15 mm, this area was estimated to be 1.875 × 10^−7^ m^2^. As a result, the pressure *P* applied to the sensor head was obtained by conversion from half of the weight gravity (*F*) through the equation *P* = *F*/*S*, where *S = wd* was the estimated contact area, assumed to be invariable in the range of detected pressures.

**Figure 3:**
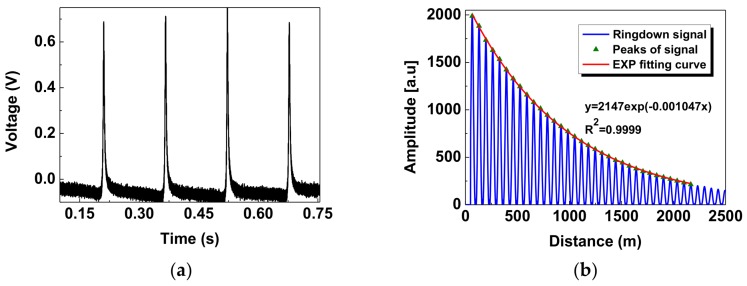
Typical differential interference signal Δ*I* without pressure applied: (**a**) the time-domain signal sampled by the DAQ; (**b**) the obtained spatial-domain cavity ringdown(CRD) signal after performing fast Fourier transform (FFT) on (**a**).

When no pressure was applied on the sensor head, a typical time-domain differential interference signal Δ*I* sampled by the DAQ is demonstrated in Figure 3a, and throughthe simple transform of the FFT algorithm, the obtained CRD decay transient in the spatial domain is shown in Figure 3b. In order to eliminate the influence of the residual DC component caused by the incomplete cancellation of the BD, the CRD signal had been set to zero in the initial distance, which ranged from 0 to 50 m. The ringdown pulses could clearly be observed with a round-trip distance (i.e., the cavity length) of ~65.7 m. By extracting the peaks of the ringdown signal and then fitting these peaks with an exponential (EXP) function, the ringdown distance without pressure applied can be calculated as 955.11 m with an R-squared of 0.9999. It reveals that the signal intensity has an excellent exponential decay relationship with the distance traveled by the continuous light in the RDC.

**Figure 4:**
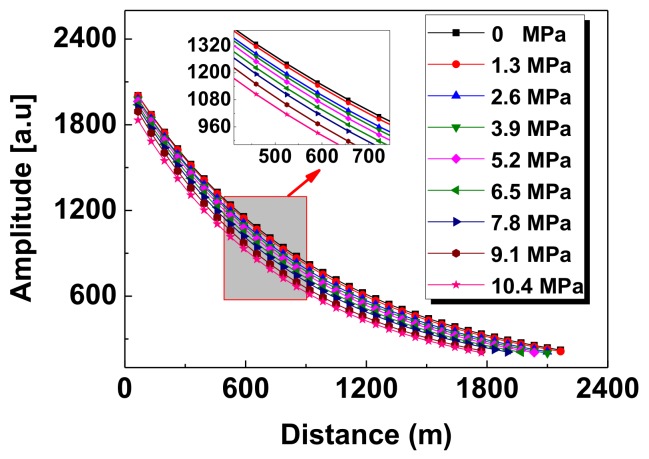
Fitted exponential decay curves under different pressures.

**Figure 5:**
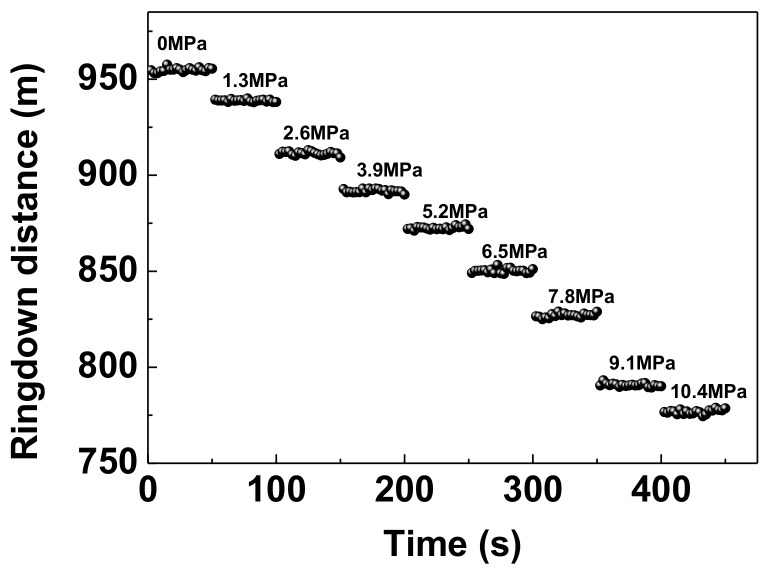
The ringdown distance responses to pressures applied on the sensor.

**Figure 6:**
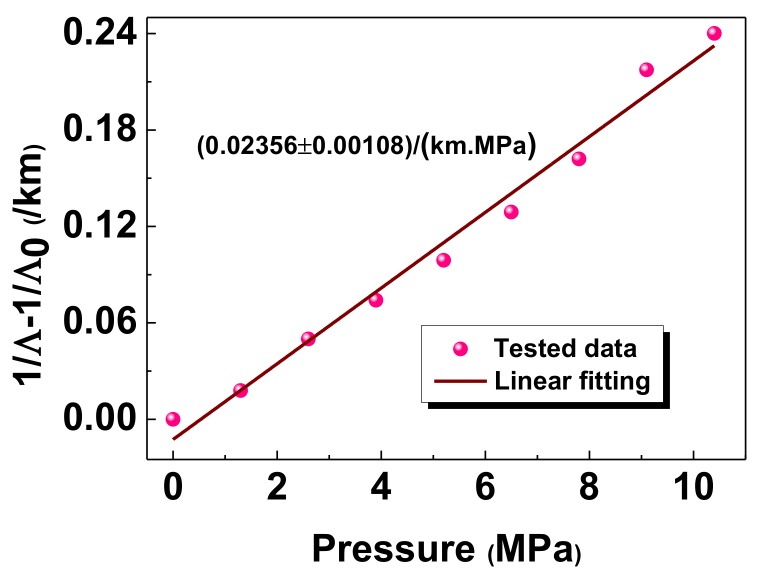
The relationship between the ringdown distance and the pressure.

We further measured spatial-domain ringdown signals with the pressures applied on the sensor head, when the quality of the applied weights was increased from 0 to 400 g at increments of 50 g. This means that the corresponding pressure applied ranged from 0 to 10.4 MPa at steps of 1.3 MPa. Through nearly the same processing method as described in Figure 3, the obtained exponential decay curves are shown in Figure 4. Each decay curve represents an average of 20 ringdown events, which is distinguishable, especially in the inset of Figure 4. We can observe that the ringdown signal decays faster as the pressure increases. This also can be confirmed by the on-line responses of the ringdown distances to the pressures applied on the sensor head, as shown in Figure 5. Each distinctive step corresponds to a different applied pressure. The averaged ringdown distance measured by FSI-FCRD decreased from 955.50 m at 0 MPa to 776.81 m at 10.4 MPa. The linearity of the sensor response was examined by use of the data in Figure 5. Figure 6 clearly illustrates the relationship between the term (1/Λ – 1/Λ_0_) and the pressure applied. The variation (1/Λ – 1/Λ_0_) increases with the increase of the applied pressure. By linear fitting, the R-squared of 0.98342 indicates that the variation of ringdown distance is a linear function of the pressure, which agrees with the analysis result of Equation (5). The slope of the fitted straight line suggests that the measurement sensitivity is 0.02356/(km∙MPa) with a standard error of 0.00108/(km∙MPa). However, one must notice that, according to Equation (5) and the contact area *S* = *wd*, the sensitivity of the sensor is proportional to the interaction length squared (*d*^2^), and thus, it can be improved by increasing the interaction length *d* of the sensor head. For instance, in our experiments, when the interaction length of the sensor was lengthened to 25 mm, the measured sensitivity increased to 0.07087/(km∙MPa), which was consistent with the theoretical value of 0.06544/(km∙MPa). The small deviation was caused by the fact that the gravity centers of the applied weights deviated from the geometric center of the contact area.

**Figure 7:**
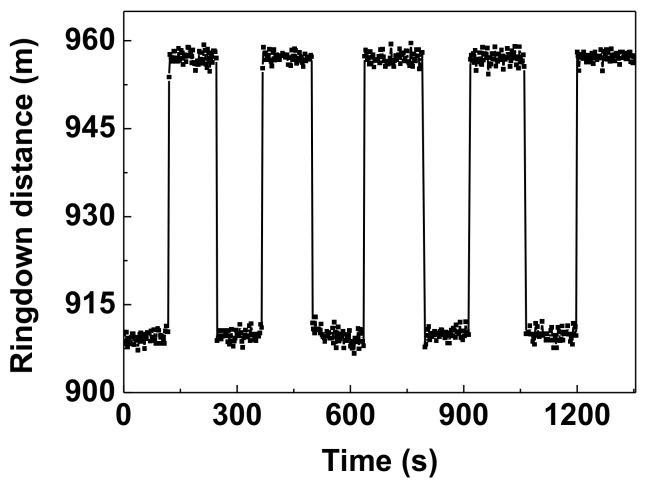
The repeated measurement results with 2.6-MPa pressure unloaded and loaded.

Figure 7 demonstrates the ringdown response to 2.6-Mpa pressure unloaded and loaded on the pressure sensor head. The up and down steps show that the sensor has a good repeatability. Additionally, each data point in Figure 7 came from one measurement of the ringdown event, which took ~2.5 s. By dividing the experimental time by the number of all the data points in [21,30] respectively, the time taken can be calculated as ~4.2 s and ~8.6 s. Therefore, the proposed sensor also has a faster pressure response.

**Figure 8:**
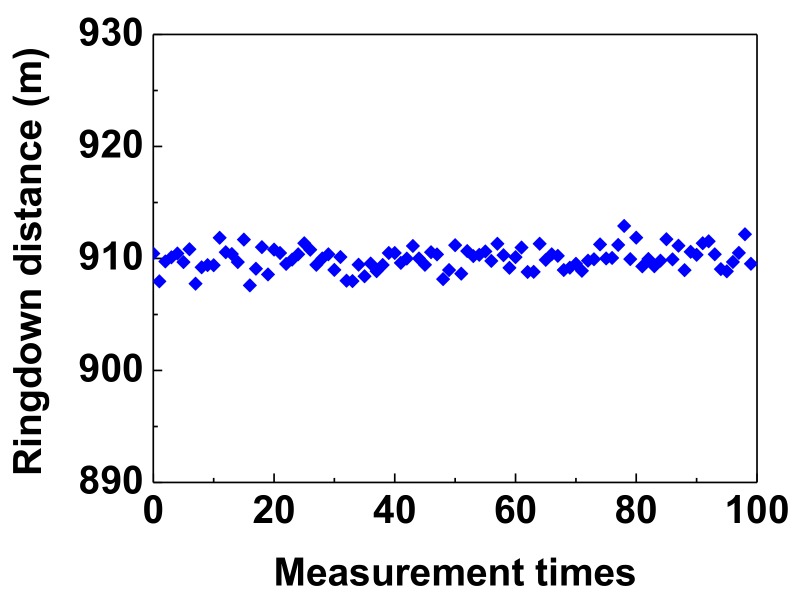
Typical testing result of the ringdown baseline stability of the pressure system.

In order to evaluate the stability of the system, the ringdown baseline stability, $\delta \Lambda /\bar{\Lambda }$, where *δ*Λ is the standard deviation and $\bar{\Lambda }$ is the averaged ringdown distance, was defined as similar to that of the literature [19]. The measurement of the ringdown distance was repeated 100 times under the same constraints (e.g., the pressure of 2.6 MPa) and the results were shown in Figure 8. The averaged ringdown distance was 909.99 m, and the obtained standard deviation was 1.02 m, much smaller than the spatial resolution of the FSI system. Therefore, the ringdown baseline stability of the FSI-FCRD system was 0.1%, which was as good as the stability reported in [19]. Compared with the baseline stabilities of ~1% reported in the literature [21,31,32], the better stability of the system is mainly due to the fact that the FSI-FCRD scheme has the nature of same-path interference, and it employs differential detection to eliminate DC components of the two interference lightbeams. The better stability of the systemis also due to the unwarped status or noninstrinsic fiber interaction in the system, neither a micro-bend fiber [21] nor a fiber endgap/taper fabricated as the sensor head in the RDC [31,32]. According to the pressure sensitivity mentioned above and Equation (7), the pressure resolution of the system for pressure measurement was determined to be 0.05 MPa.This is better than those reported in [19,30], because the pressure detection limits can be calculated as ~0.07 MPa by using the equation $P_{m}\approx \delta \tau /(k{\bar{\tau }}^{2})$, similar to Equation (7), where $\bar{\tau }$ is the averaged value of the CRD times, δτ is the corresponding one standard deviation, and *k* is the pressure sensitivity obtained on the basis of the parameters measured in the texts. Moreover, the detection limit of our proposed sensor can be further improved by increasing the pressure sensitivity.

## 4. Conclusions

A novel method of developing optical fiber pressure sensors by use of a continuous-wave FCRD scheme based on FSI is demonstrated. With this FSI-FCRD technique, pressure measurements were achieved in a spatial domain by measuring ringdown distances. The sensing system consists of a continuous-wave broadband light source, an acousto-optic modulator, four fiber couplers, a fraction of single-mode fiber, and a slow detector. The sensor’s performance in the issues of linear response, sensitivity, repeatability, and stability was investigated experimentally by applying weights to the sensor head. A sensitivity of 0.02356/(km∙MPa) was attained in the range of 0–10.4 MPa. Furthermore, through adjusting the interaction length of the sensor head in the fiber RDC, the sensing system allows for tailoring of sensitivity. In the repetitive test, the obtained measurement error was 0.1%, corresponding to the minimum detectable pressure of 0.05 MPa, which is better than the detection limits reported in [19,30]. Additionally, the FSI-FCRD pressure sensor has a rapid response time, typically of less than 3 s. The results indicate that the proposed sensor is low-cost, highly sensitive, and highly stable, and it exhibits the application potential for pressure measurements in aerospace, petrochemical industries, ice loading on bridge piers, smart materials, etc.
